# Impact of intravitreal aflibercept dosing regimens in treatment-naïve patients with neovascular age-related macular degeneration: 2-year results of RAINBOW

**DOI:** 10.1186/s12886-020-01468-z

**Published:** 2020-05-25

**Authors:** Michel Weber, Marcel Dominguez, Florence Coscas, Céline Faure, Stéphanie Baillif, Laurent Kodjikian, Salomon-Yves Cohen

**Affiliations:** 1 CHU Hôtel-Dieu, 44000 Nantes, France; 2 Centre Rétine Galien, Bordeaux, France; 3 Centre Odéon, Paris, France; 4 Clinique Saint Martin, Ramsay Générale de Santé, Caen, France; 5 CHU Nice, Hôpital Pasteur 2, Nice, France; 6 Croix-Rousse University Hospital, Hospices Civils de Lyon, University of Lyon I, Lyon, France; 7 CNRS UMR Mateis, Villeurbanne, France; 8 Centre d’Imagerie Et de Laser, Paris, France

**Keywords:** Intravitreal aflibercept, Neovascular age-related macular degeneration, France, Real-world, Observational

## Abstract

**Background:**

To review treatment outcomes from real-world data of patients with neovascular age-related macular degeneration (nAMD) treated with intravitreal aflibercept (IVT-AFL) injection.

**Methods:**

RAINBOW (ClinicalTrials.gov, NCT02279537) is an ongoing, observational, 4-year study to monitor the effectiveness and safety of IVT-AFL in patients with nAMD in clinical practice in France. Treatment-naïve patients diagnosed with nAMD who had been prescribed IVT-AFL by their treating physician were eligible. The regimens of interest were regular treatment interval cohort (patients who received three initial monthly IVT-AFL injections followed by regular injections every 2 months) and two irregular treatment interval cohorts (with and without three initial monthly injections). Here we describe results at 24 months in patients according to IVT-AFL treatment regimen.

**Results:**

The mean change in best-corrected visual acuity (BCVA) with IVT-AFL from baseline to 24 months was + 3.0 letters in the overall population (*P* < 0.05 vs baseline). The mean change was positive for the regular and irregular treatment interval cohorts with initial doses (+ 4.9 and + 4.0 letters, respectively; *P* < 0.05 vs baseline) and negative for the irregular treatment interval cohort without initial doses (− 2.5 letters; *P* = 0.365 vs baseline) at 24 months. The mean overall number of IVT-AFL injections over 12 and 24 months was 6.0 and 8.8, respectively. The most common ocular adverse events were lack of efficacy (6.3%), vitreous floaters (2.7%), and increased lacrimation (1.7%).

**Conclusions:**

In the real-world RAINBOW study, visual outcomes observed at 24 months were consistent with results from the primary endpoint at 12 months. In this study, treatment-naïve patients who received three initial IVT-AFL doses and regular IVT-AFL treatment over the first 24 months experienced better visual outcomes than patients who received no initial doses and an irregular treatment regimen.

**Trial registration:**

www.ClinicalTrials.gov (NCT02279537). Registered 29 October 2014.

## Background

There are two forms of age-related macular degeneration (AMD), the neovascular and the dry forms [1]. Anti-vascular endothelial growth factor (anti-VEGF) agents, such as intravitreal aflibercept (IVT-AFL) and ranibizumab, are available for the treatment of neovascular age-related macular degeneration (nAMD), and the goal of disease management beyond the first year is to maintain or improve functional and anatomical gains while minimizing the burden on patients of clinic visits and injections [2]. Although rapid visual and anatomic improvements can be achieved in the first year of anti-VEGF treatment, regression to baseline after initial gains is not uncommon [2]. Findings from the VEGF Trap-Eye Investigation of Efficacy and Safety in Wet AMD (VIEW) studies showed that, in the second year of treatment, the dosing interval for IVT-AFL can be adjusted according to the patient’s response to treatment, without clinically meaningful loss of visual gains [3, 4].

Before IVT-AFL was authorized in Europe in 2012, anti-VEGF treatments for nAMD in France were administered as needed (pro re nata) [5]. The introduction of IVT-AFL led to changes in standard clinical practice from reactive to proactive treatment protocols/regimens. As clinicians became proficient with newly available anti-VEGF treatments for nAMD, injection intervals varied substantially, as did adherence to the indicated initiation of IVT-AFL with three initial monthly doses [6]. In the Real Life of intravitreal Aflibercept In FraNce: oBservatiOnal study in Wet AMD (RAINBOW) study, patients treated with IVT-AFL for nAMD were expected to receive three initial monthly injections followed by injections every 2 months for the first 12 months, with extensions based on visual and anatomic outcomes thereafter [4]. The purpose of this study was not to identify differences in clinical practices before and after any changes in approved dosing for IVT-AFL, but rather to describe clinically led variations in treatment practices in France and understand any impact of those on patient outcomes.

Currently, there is limited real-world evidence demonstrating how nAMD is managed in daily clinical practice in France. Real-world evidence can complement clinical trial data by providing information on the effectiveness of a treatment under real-world conditions that can support clinical management decisions and improve patient outcomes [7, 8]. Here, we report results from the second year of the RAINBOW, an ongoing study collecting effectiveness and safety data from patients with nAMD treated with IVT-AFL in clinical practice in France.

## Methods

### Study design

RAINBOW (NCT02279537) is an observational, 4-year study designed to monitor the effectiveness and safety of IVT-AFL in patients with nAMD in clinical practice. Patients were enrolled from 55 centers across France consisting of private, hospital and mixed-type clinical settings. The present analysis reports 24-month outcomes for patients followed up for a period of 4 years or until discontinuation. The data collection period started in October 2014. Data from patients who started IVT-AFL treatment between January 2 and October 13, 2014, were retrospectively collected and then prospectively collected from October 14, 2014, onwards. Data are collected during the initial visit and the routine follow-up visits.

### Procedures

As RAINBOW is an observational study, there was no study pre-specified retreatment criteria, it was at the investigator’s discretion based on clinical expertise and routine medical practice to determine when retreatment was needed.

### Participants

Patients aged ≥18 years with a diagnosis of nAMD who were treatment naïve to any anti-VEGF agent or macular laser in the study eye, which was defined as the worst-seeing eye of each patient, but the second eye was also considered if it was treatment naïve. Patients were excluded if they had another retinal disease (i.e. diabetic retinopathy, diabetic macular oedema, myopic choroidal neovascularization, retinal vein occlusion, central serous chorioretinopathy, or angioid streaks) or if they were participating in any other interventional study.

### Endpoints

The primary endpoint was the change in best-corrected visual acuity (BCVA) from baseline to Month 12 as assessed by the Early Treatment Diabetic Retinopathy Study (ETDRS) protocol or a visual logarithmic scale. These results have been previously published [6]. Results at 24 months in patients according to IVT-AFL treatment regimen are presented here. Secondary outcomes included the percentage of patients who gained more than 0, 5, 10, or 15 letters, or lost more than 15 letters at 24 months; and the proportion of patients with BCVA over and inclusive of 70 letters at 24 months. All adverse events (AEs) reported after the first injection of IVT-AFL, and up to 30 days after the last IVT-AFL injection, were documented.

### Statistical analysis

Sample size calculations indicated that 600 patients needed to be enrolled in the RAINBOW study to achieve 390 usable data sets at Month 48. These estimates were based on the VIEW studies, using a 10% annual dropout rate. Visual acuity analyses were based on the full analysis set (FAS), which included patients who had documented visual acuity and anatomic assessments (in the study eye) at baseline and at least once during follow-up. The FAS-targeted group had documented visual acuity assessments in the study eye at baseline and at Month 24. The safety analysis set (SAS) comprised data from patients who received at least one IVT-AFL injection.

This 24-month analysis reports the effectiveness and safety of IVT-AFL in patients with documented visual acuity assessments at baseline and Month 24 (FAS-targeted group), stratified by IVT-AFL regimen as follows: (1) regular treatment interval cohort: patients who received three initial monthly (− 1/+ 2 weeks) IVT-AFL doses and then IVT-AFL every 2 months (− 3/+ 4 weeks) with ≥6 injections during the first 12 months; (2) irregular treatment interval cohort with three initial doses: patients who received IVT-AFL every < 2 or > 2 months with three initial doses over the first 12 months; or (3) irregular treatment interval cohort without three initial doses: including patients who received IVT-AFL every < 2 or > 2 months without three initial doses over the first 12 months. After the first 12 months, treatment frequency and modality for all cohorts was at the discretion of the treating physician and in accordance with approved local prescribing information. The statistical analysis was performed with the software package SAS, release 9.4 (SAS Institute Inc., Cary, NC, USA).

## Results

### Patients

**Fig. 1:**
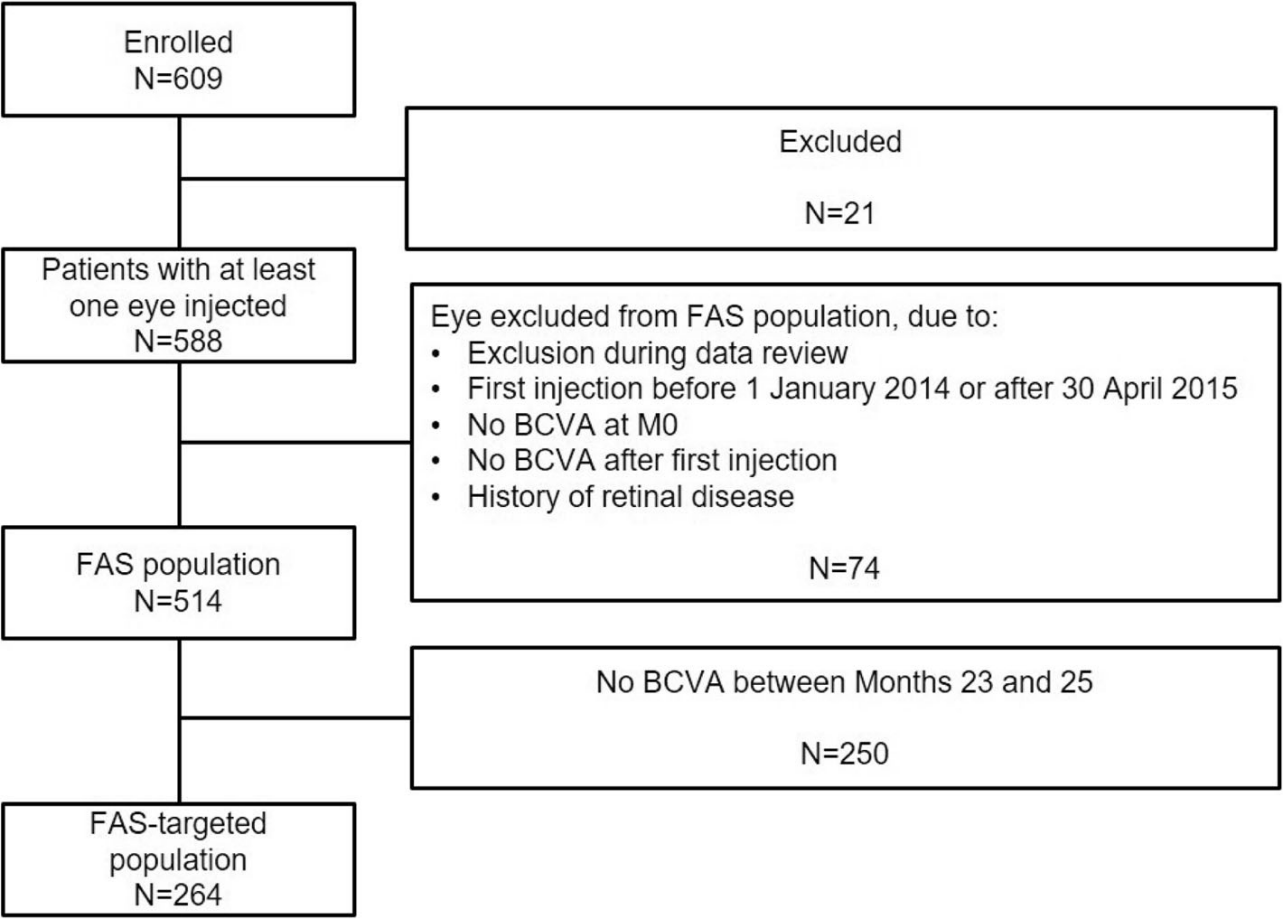
Patient disposition during the study. *BCVA* best-corrected visual acuity, *FAS* full analysis set, *M0* Month zero

Data from 514 patients who had BCVA scores at baseline and at least one follow-up assessment were included in this 24-month analysis (the FAS, or overall population). A total of 102 patients were included in the regular treatment interval cohort, and 268 and 60 patients were included in the irregular treatment interval cohorts with and without initial doses, respectively. Overall, 264 patients had documented visual acuity assessments in the study eye at baseline and Month 24 (the FAS-targeted population). Safety data were analysed from 588 patients (SAS) (Fig. 1). Patient baseline demographics and characteristics (FAS and FAS-targeted populations) are presented in Table 1**.** The mean (standard deviation; SD) age at enrolment was 79.6 (7.9) years and the mean (SD) duration of nAMD was 1.4 (8.7) months.

**Table 1:** Patient demographic and baseline characteristics

Characteristic	FAS population ***N*** = 514	FAS-targeted population at Months 0 and 24 ***N*** = 264
Age, years	79.6 (7.9)	79.2 (7.7)
Female, *n* (%)	315 (61.3)	163 (61.7)
Study eye, *n* (%)		
Right	281 (54.7)	149 (56.4)
Left	233 (45.3)	115 (43.6)
Duration of nAMD, months	1.4 (8.7)	1.8 (11.0)
BCVA (letters) score at month 0	56.3 (18.6)	57.3 (17.9)
BCVA (letters) categories, *n* (%)		
< 50	143 (27.8)	65 (24.6)
50–55	64 (12.5)	35 (13.3)
55–70	151 (29.4)	78 (29.5)
≥ 70	156 (30.4)	86 (32.6)
Intraocular pressure, mm Hg (*n* = 262)	14.9 (3.1)	–
Diabetes, *n* (%)	43 (8.4)	–
Hypertension, *n* (%)	196 (38.1)	–
Cardiovascular diseases, *n* (%)	87 (16.9)	–

Mean (SD) unless otherwise stated

*BCVA* best-corrected visual acuity, *FAS* full analysis set, *nAMD* neovascular age-related macular degeneration, *SD* standard deviation

### Injections and visits

Over 12 and 24 months, the mean number of IVT-AFL injections was 6.0 and 8.8, respectively; the difference in mean number indicated that there were fewer injections in the second year (Table 2). The interquartile range (Q1; Q3, 25–75% of injections) for the regular cohort was 8.0 to 13.0 injections, while for the irregular cohorts with and without initial doses was 5.0 to 12.0 and 4.0 to 11.0, respectively (Table 2). The mean number of clinic visits over 12 and 24 months was 9.3 and 15.1, respectively (Table 2). The interquartile range (Q1; Q3, 25–75% of visits) for the regular cohort was 13.0 to 19.0, while for the irregular cohorts with and without initial doses was 14.0 to 20.0 and 12.0 to 17.0 visits (Table 2).

**Table 2:** IVT-AFL injections and visits over 12 and 24 months of treatment

	Overall population (FAS; ***n*** = 514)	Regular cohort (***n*** = 102)	Irregular cohort with initial doses (***n*** = 268)	Irregular cohort without initial doses (***n*** = 60)
**IVT-AFL injections**				
Mean (SD) over 12 Months	6.0 (2.1)	7.2 (0.8)	6.1 (2.2)	5.2 (1.8)
Mean (SD) over 24 Months	8.8 (4.3)	10.6 (2.8)	9.3 (4.6)	7.8 (3.7)
Min; max	1.0; 23.0	6.0; 17.0	3.0; 23.0	1.0; 16.0
Median	8.0	11.0	9.0	8.0
Q1; Q3	5.0; 12.0	8.0; 13.0	5.0; 12.0	4.0; 11.0
**Visits**				
Mean (SD) over 12 Months	9.3 (2.3)	9.5 (1.8)	10.1 (1.9)	8.8 (1.8)
Mean (SD) over 24 Months	15.1 (5.1)	15.9 (4.2)	16.8 (4.2)	14.8 (3.8)
Min; max	2.0; 29.0	8.0; 27.0	6.0; 29.0	6.0; 23.0
Median	15.0	16.0	17.0	15.0
Q1; Q3	12.0; 19.0	13.0; 19.0	14.0; 20.0	12.0; 17.0

*FAS* full analysis set, *IVT-AFL* intravitreal aflibercept, *n* n numbers from the overall FAS population, Q1;Q3, interquartile range, *SD* standard deviation

### Visual outcomes

**Fig. 2:**
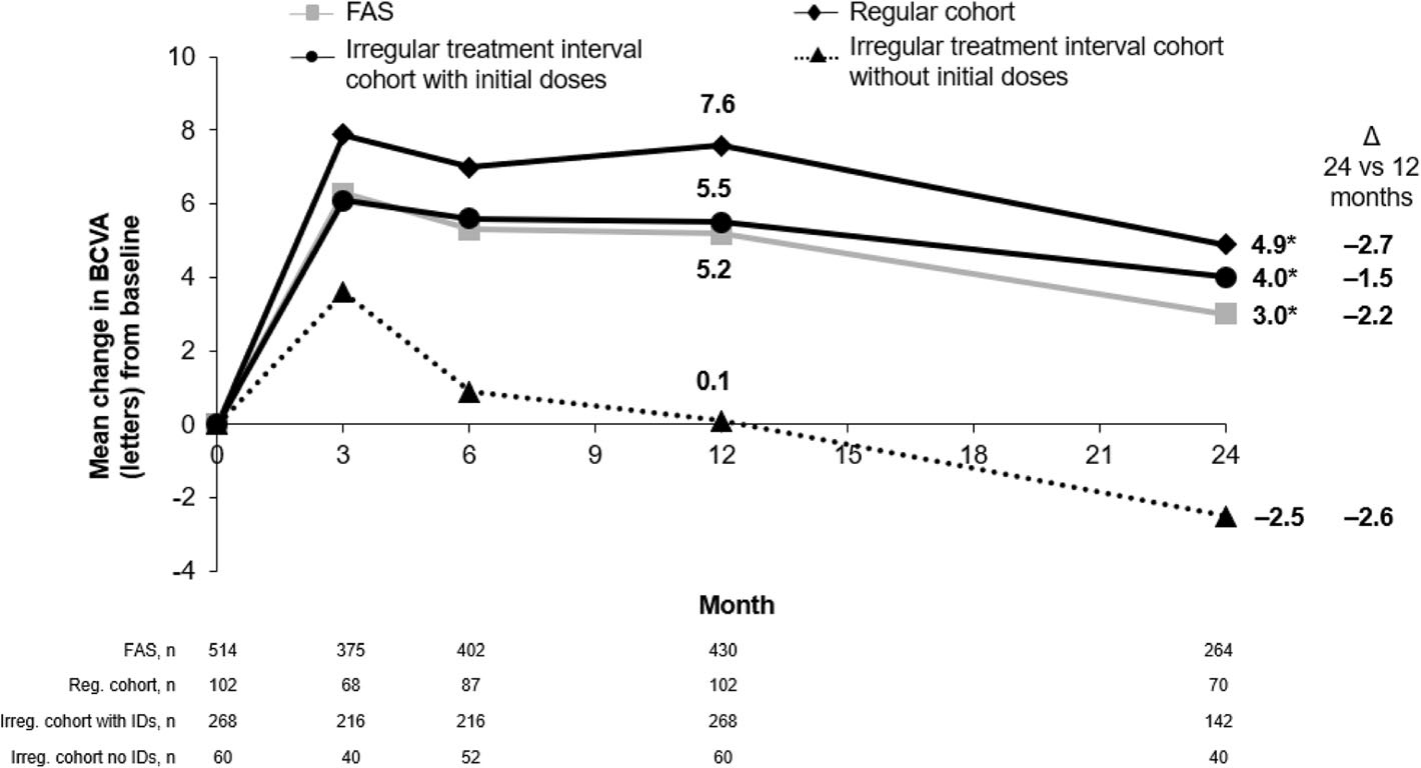
Change in visual acuity over 12 and 24 months according to intravitreal aflibercept regimen. Observed analysis. *P < 0.05 versus baseline. *BCVA* best-corrected visual acuity, *FAS* full analysis set, *IDs*, initial doses, *irreg* irregular, *reg* regular, *n* number or patients

**Fig. 3:**
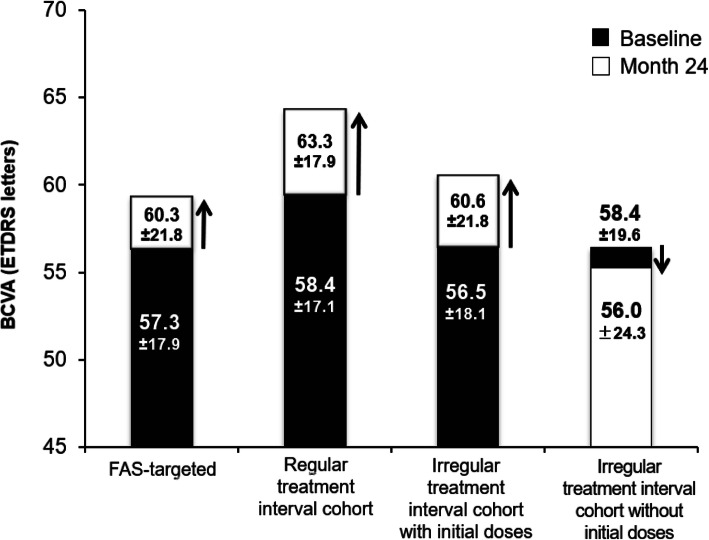
Mean BCVA score at baseline and at Month 24. FAS targeted: *n* = 264. Regular treatment interval cohort: *n* = 70. Irregular treatment interval cohort with initial doses: *n* = 142. Irregular treatment interval cohort without initial doses: *n* = 40. *BCVA* best-corrected visual acuity, *ETDRS* Early Treatment Diabetic Retinopathy Study, *FAS* full analysis set

The mean change in BCVA with IVT-AFL treatment over 24 months was + 3.0 letters in the overall population (*P* < 0.05 vs baseline). The mean change from baseline was positive for the regular and irregular treatment interval cohorts with initial doses (+ 4.9 and + 4.0 letters, respectively; *P* < 0.05 vs baseline for both) and negative for the irregular treatment interval cohort without initial doses (− 2.5 letters; *P* = 0.365) (Fig. 2). The visual gains observed with regular versus irregular IVT-AFL treatment regimens with initial doses were not statistically different (*P* = 0.571). The mean gain observed for the regular treatment interval cohort was significantly greater than the gain observed in the irregular treatment interval without initial dose (*P* = 0.036). The mean BCVA score in the overall population increased from 57.3 at baseline to 60.3 at Month 24 (Fig. 3).

**Fig. 4:**
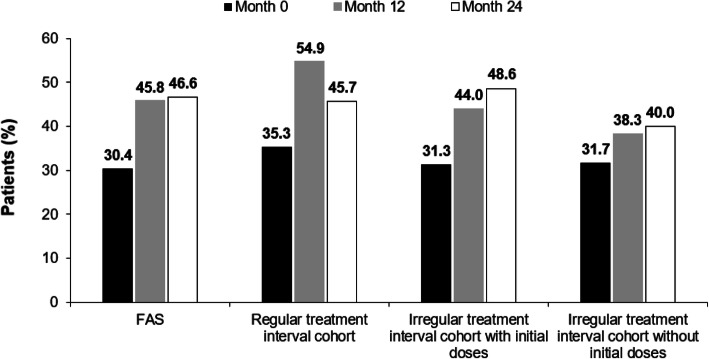
Proportion of patients with a BCVA ≥70 letters by IVT-AFL regimen. Observed analysis. Regular treatment interval cohort: *n* = 102 (month 0); *n* = 102 (month 12); *n* = 70 (month 24). Irregular treatment interval cohort with initial doses: *n* = 268 (month 0); *n* = 268 (month 12); *n* = 142 (month 24). Irregular treatment interval cohort without initial doses: *n* = 60 (month 0); *n* = 60 (month 12); *n* = 40 (month 24). *BCVA* best corrected visual acuity, *IVT-AFL* intravitreal aflibercept

The proportion of patients who experienced a gain in visual acuity letter score of ≥15 letters from the initial visit to Month 24 was 26.5% in the overall population. When broken down by treatment frequency, a gain of ≥15 letters was observed in 25.7% of the regular treatment interval cohort and 31.7% of the irregular treatment interval cohort with initial doses. The proportion of patients in the irregular treatment interval cohort without initial doses who had a gain of ≥15 letters was 10.0% (odds ratio [OR; 95% CI]): 3.95 [1.11–14.03]; *P* = 0.034, a significantly lower proportion than the regular treatment cohort. The odds for patients in the irregular treatment interval cohort to gain ≥15 letters in a 24-month period were five-fold greater than for patients in the irregular interval treatment cohort without initial doses (5.03 [1.53–16.61]; *P* = 0.008). The proportion of patients who experienced a loss in visual acuity letter score of > 15 letters from the initial visit to Month 24 was 12.1% in the overall population and 7.1% in the regular treatment interval cohorts. The proportion of patients who experienced a loss in visual acuity letter score of > 15 letters from the initial visit to Month 24 was comparable between the irregular treatment interval cohorts with initial doses (14.1%; OR [95% CI]: 0.47 [0.17–1.31]; not significant [NS]) and without initial doses (12.5%; OR [95% CI]: 0.54 [0.15–2.0]; NS), respectively. The proportion of patients in the overall population who achieved ≥70 letters according to IVT-AFL regimen is presented in Fig. 4.

### Safety

Overall, 38.4% (n/*N* = 226/588) of patients experienced at least one treatment-emergent adverse event (TEAE) and 10% (*n* = 59) experienced at least one treatment-related TEAE (Table 3). The most common ocular AEs were lack of efficacy (6.3%), vitreous floaters (2.7%), and lacrimation increased (1.7%). The most common non-ocular AEs included a product-use issue (3.1%), bronchitis (1.9%), fall (1.2%), and malaise (1.0%). Serious TEAEs were reported in 10.9% of patients. Five deaths were recorded during the study; no deaths were considered related to treatment.

**Table 3:** Safety outcomes at Month 24

Safety population (*N* = 588)	Total ***n*** (%)
Any TEAE	226 (38.4)
Any treatment-related TEAE	59 (10.0)
Ocular	54 (9.2)
Non-ocular	5 (0.9)
Any ocular TEAE	136 (23.1)
Most common ocular AE	
Lack of efficacy	37 (6.3)
Vitreous floaters	16 (2.7)
Lacrimation increased	10 (1.7)
Visual acuity reduced	9 (1.5)
Retinal pigment epithelium detachment	9 (1.5)
Any non-ocular TEAE	123 (20.9)
Most common non-ocular AE	
Inappropriate schedule of drug administration	18 (3.1)
Bronchitis	11 (1.9)
Fall	7 (1.2)
Malaise	6 (1.0)
Influenza	5 (0.9)
Discontinuation due to TEAE	72 (12.2)
Discontinuation due to treatment-related TEAE	40 (6.8)
Any serious TEAE	64 (10.9)
Ocular	9 (1.5)
Non-ocular	59 (10.0)
Serious TEAE (> 0.5%)	
Transient ischemic attack	4 (0.7)
Death	5 (0.9)

*AE* adverse events; *TEAE* treatment-emergent adverse event

## Discussion

RAINBOW is an ongoing study investigating the effectiveness and safety of IVT-AFL for the management of treatment-naïve patients with nAMD in real-life clinical practice in France. In the present analysis, visual improvements were observed at Month 24 in patients with nAMD following IVT-AFL treatment, with an increase from baseline in BCVA of + 3.0 letters in the overall population. Notably, patients who received three initial monthly doses and regular IVT-AFL treatment experienced better visual outcomes over 24 months than patients with irregular treatment without initial doses. Visual gains observed with regular versus irregular IVT-AFL treatment with initial doses were not statistically different. A significant mean change from baseline in BCVA was observed in the regular and irregular treatment interval cohorts receiving initial doses, + 4.9 and + 4.0 letters, respectively, while the irregular treatment interval cohort without initial doses of IVT-AFL experienced a change of − 2.5 letters at 24 months. The findings from this analysis at 24 months are consistent with results from the initial 12-month analysis [6] and highlight the importance of three initial monthly IVT-AFL treatments to stabilize the disease and maintain visual outcomes. The change from baseline in BCVA in this analysis was not as large as in the VIEW clinical trials at Week 96 (+ 7.6 letters) [3]. In VIEW the percentage of patients gaining ≥15 letters was 33.4%, and the percentage of patients maintaining visual acuity (losing < 15 letters) was 92.4% at Week 96 in the IVT-AFL 2 mg group. It is important to note that in the VIEW studies, in Year 1 patients received three initial doses and bimonthly injections, while in Year 2 patients received an as-needed injection regimen with defined retreatment criteria and mandatory dosing at least every 12 weeks [3]. In the VIEW study, patients received a mean of 11.2 IVT-AFL injections over the 96-week period, with fewer injections in the second year (4.2 injections from Week 52 to 96) [3]. In comparison, over the analysis period in the present study, the mean number of IVT-AFL injections was 8.8 and, similar to the VIEW studies, there were fewer injections in the second year. However, in the VIEW studies patients exhibited a more severe disease (mean visual acuity at baseline = 53.6 [13.5] letters in the patients treated with IVT-AFL 2 mg bimonthly) [3] than in the RAINBOW study.

Real-world evidence from the use of IVT-AFL for the treatment of nAMD is also being collected in other countries. PERSEUS (Prospective Non-intERventional Study to asSEss the Effectiveness of Aflibercept in roUtine Clinical Practice in patientS With Wet Age-related Macular Degeneration) is a 24-month prospective observational cohort study conducted in hospitals and medical centers in Germany among treatment-naïve or previously treated patients with nAMD. Following IVT-AFL, there was a significant mean visual acuity gain at 1 year of + 6.1 letters in the population with regular treatment intervals for IVT-AFL compared with + 1.5 letters in those with irregularities in their treatment regimen [9]. An observational database study in treatment-naïve patients with nAMD from centers in Australia, New Zealand, and Switzerland was conducted by the Fight Retinal Blindness (FRB) group. Patients completed 2 years of proactive IVT-AFL treatment according to a treat-and-extend regimen. Over 2 years of treatment, a significant increase in mean visual acuity was reported (+ 6.0 letters). In the FRB study, from the first to the second year of treatment there was a decrease in the mean number of injections (7.8 vs 5.7) and visits (8.7 vs 6.5) for eyes completing 2 years of treatment [10]. In the present study, the poor visual acuity in the irregular treatment interval cohort without initial doses appears to be more likely related to few patients (10%) achieving gains ≥15 letters, patients losing > 15 letters (12.5%) and fewer treatment injections and visits over the 24 months than other treatment groups. The findings from the present study add to the real-world evidence supporting the continued use of IVT-AFL through 24 months.

Safety findings from the RAINBOW study at 24 months were consistent with the known safety profile of IVT-AFL in patients with nAMD [3, 11]. Five deaths were recorded during the study; none were considered related to treatment. The incidence of ocular AEs was lower in the present study than in VIEW studies, which may be due to possible underreporting in an observational study compared with a randomized study.

Due to the observational design of the RAINBOW study there are a number of inherent limitations that must be recognized when interpreting these findings. The use of a variety of charts to assess visual acuity may have introduced bias, especially when evaluating the number of letters gained or lost after treatment. Furthermore, the limitations of analysing the evolution of visual acuity in relation to the number of injections only must be recognized. Disease-related evolution of nAMD can occur that may not be directly influenced by treatment, even if treatment is optimal. In addition, these findings reflect real-life clinical practice in France and may not be generalisable across countries.

## Conclusions

Findings from the RAINBOW study at 24 months were consistent with results from the primary endpoint at 12 months and highlight the importance of three initial monthly IVT-AFL injections on visual outcomes. Notably, treatment-naïve patients who received three initial monthly IVT-AFL doses followed by regular treatment over the first 12 months experienced better visual outcomes at 24 months compared with irregular treatment without initial doses. The overall safety profile was consistent with previous studies of IVT-AFL. The results of this 24-month analysis of the 4-year RAINBOW study were only slightly lower than those of randomized studies, such as VIEW, and demonstrate that IVT-AFL (with initial doses) offers treatment effectiveness in real-world practice.

## Data Availability

Availability of the data underlying this publication will be determined according to Bayer’s commitment to the EFPIA/PhRMA “Principles for responsible clinical trial data sharing”. This pertains to scope, time point and process of data access. As such, Bayer commits to sharing upon request from qualified scientific and medical researchers patient-level clinical trial data, study-level clinical trial data, and protocols from clinical trials in patients for medicines and indications approved in the United States (US) and European Union (EU) as necessary for conducting legitimate research. This applies to data on new medicines and indications that have been approved by the EU and US regulatory agencies on or after January 01, 2014. Interested researchers can use www.clinicalstudydatarequest.com to request access to anonymized patient-level data and supporting documents from clinical studies to conduct further research that can help advance medical science or improve patient care. Information on the Bayer criteria for listing studies and other relevant information is provided in the Study sponsors section of the portal. Data access will be granted to anonymized patient-level data, protocols and clinical study reports after approval by an independent scientific review panel. Bayer is not involved in the decisions made by the independent review panel. Bayer will take all necessary measures to ensure that patient privacy is safeguarded.

## Abbreviations

AE: Adverse event
AMD: Age-related macular degeneration
anti-VEGF: Anti-vascular endothelial growth factor
ARVO: Association for Research in Vision and Ophthalmology
BCVA: Best-corrected visual acuity
CCTIRS: Comité Consultatif sur le Traitement de l’Information en Matière de Recherche dans le Domaine de la Santé
CNIL: Commission Nationale de l’Informatique et des Libertés
EFPIA: European Federation of Pharmaceutical Industries and Associations
ETDRS: Early Treatment Diabetic Retinopathy Study
EU: European Union
EURETINA: European Society of Retina Specialists
FAS: Full analysis set
FRB: Fight Retinal Blindness
IVT-AFL: Intravitreal aflibercept
M0: Month zero
nAMD: Neovascular age-related macular degeneration
n: Number of patients
NS: Not significant
OR: Odds ratio
PERSEUS: Prospective Non-intERventional Study to asSEss the Effectiveness of Aflibercept in roUtine Clinical Practice in patientS With Wet Age-related Macular Degeneration
PhRMA: Pharmaceutical Research and Manufacturers of America
Q: Interquartile range
RAINBOW: Real Life of intravitreal Aflibercept In FraNce: oBservatiOnal study in Wet AMD
SAS: Safety analysis set
SD: Standard deviation
SFO: Société Française d’Ophtalmologie
TEAE: Treatment-emergent adverse event
UK: United Kingdom
US: United States
VIEW: VEGF Trap-Eye Investigation of Efficacy and Safety in Wet AMD
